# Tracking Endogenous Amelogenin and Ameloblastin *In Vivo*

**DOI:** 10.1371/journal.pone.0099626

**Published:** 2014-06-16

**Authors:** Jaime Jacques, Dominique Hotton, Muriel De la Dure-Molla, Stephane Petit, Audrey Asselin, Ashok B. Kulkarni, Carolyn Winters Gibson, Steven Joseph Brookes, Ariane Berdal, Juliane Isaac

**Affiliations:** 1 Laboratory of Molecular Oral Pathophysiology, INSERM UMRS 1138, Team Berdal, Cordeliers Research Center, Pierre and Marie Curie University - Paris 6, Paris Descartes University - Paris 5, Paris, France; 2 UFR d'Odontologie, Paris Diderot University - Paris 7, Paris, France; 3 Unit of Periodontology, Department of Stomatology, University of Talca, Talca, Chile; 4 Center of Rare Malformations of the Face and Oral Cavity (MAFACE), Hospital Rothschild, AP-HP, Paris, France; 5 Functional Genomics Section, Laboratory of Cell and Developmental Biology, National Institute of Dental and Craniofacial Research, National Institutes of Health, Bethesda, Maryland, United States of America; 6 Department of Anatomy and Cell Biology, University of Pennsylvania School of Dental Medicine, Philadelphia, Pennsylvania, United States of America; 7 Department of Oral Biology, School of Dentistry, University of Leeds, United Kingdom; 8 Laboratory of Morphogenesis Molecular Genetics, Department of Developmental and Stem Cells Biology, Institut Pasteur, CNRS URA 2578, Paris, France; University of Pécs Medical School, Hungary

## Abstract

Research on enamel matrix proteins (EMPs) is centered on understanding their role in enamel biomineralization and their bioactivity for tissue engineering. While therapeutic application of EMPs has been widely documented, their expression and biological function in non-enamel tissues is unclear. Our first aim was to screen for amelogenin (AMELX) and ameloblastin (AMBN) gene expression in mandibular bones and soft tissues isolated from adult mice (15 weeks old). Using RT-PCR, we showed mRNA expression of AMELX and AMBN in mandibular alveolar and basal bones and, at low levels, in several soft tissues; eyes and ovaries were RNA-positive for AMELX and eyes, tongues and testicles for AMBN. Moreover, in mandibular tissues AMELX and AMBN mRNA levels varied according to two parameters: 1) ontogenic stage (decreasing with age), and 2) tissue-type (*e.g.* higher level in dental epithelial cells and alveolar bone when compared to basal bone and dental mesenchymal cells in 1 week old mice). *In situ* hybridization and immunohistodetection were performed in mandibular tissues using AMELX KO mice as controls. We identified AMELX-producing (RNA-positive) cells lining the adjacent alveolar bone and AMBN and AMELX proteins in the microenvironment surrounding EMPs-producing cells. Western blotting of proteins extracted by non-dissociative means revealed that AMELX and AMBN are not exclusive to mineralized matrix; they are present to some degree in a solubilized state in mandibular bone and presumably have some capacity to diffuse. Our data support the notion that AMELX and AMBN may function as growth factor-like molecules solubilized in the aqueous microenvironment. In jaws, they might play some role in bone physiology through autocrine/paracrine pathways, particularly during development and stress-induced remodeling.

## Introduction

The specific properties of mineralized tissues result from their unique extracellular matrix (ECM) composition. ECM has multiple effects on the biological behavior of skeletal cells and extracellular mineralization. As illustrated by the SIBLING family of proteins [1], ECM proteins not only provide template for ordered nucleation and crystal growth [2] but also control fate and activity of cells responsible for odontogenesis and cells regulating bone formation and turn-over. The organic matrix of bone, dentin and cementum is based on type I collagen associated with number of bone/tooth non-collagenous proteins [3]. In contrast, enamel is composed of specific enamel matrix proteins (EMPs) such as amelogenin (AMELX) and ameloblastin (AMBN). Contrary to bone, dentin or cementum ECM proteins, EMPs are ephemeral; after their secretion in enamel ECM and their aggregation into nanospheric structures, AMELX and AMBN are subject to proteolytic processing [4], [5].

In recent years, EMPs have been identified in root epithelial cells [6] and non-enamel dental and bone cells [7]–[12]. Presence of EMPs RNA/proteins were also reported during early tooth development at the pre-mineralization stage [13] and in organs neither related to ectodermal appendages nor mineralized tissues, such as brain [14]–[16]. Based on these observations, AMELX [14] and AMBN [17] might be functional in non-enamel tissues.

EMPs exhibit cell signaling properties that impact on a wide range of cell activities. A commercially available enamel matrix derivative (EMD) is used for periodontal regeneration as well as epidermal wound healing (for review, [17]). More specifically, using recombinant AMELX and AMBN and transgenic mice that overexpressed EMPs and their splicing forms, previous studies have demonstrated that EMPs control cell adhesion, proliferation, polarity, commitment, differentiation and act on key-cellular pathways [18]–[22]. To date, nearly all the cells of dental-periodontal, epidermal and bone compartments have been found to respond to EMPs (for review, [23]). Transgenic mouse studies indicated that osteoblast and osteoclast cell activities are influenced by AMELX and AMBN [7], [24], [25]. Thus, an extensive number of investigations have documented *in vitro* and *in vivo* cell responses to under- or over-expression of EMPs, knockdown of EMPs, ectopic expression or addition of specific recombinants, synthetic peptides or EMD fractions. Herein we describe the endogenous expression of both AMELX and AMBN in mandibular bone and soft tissues. We also report the potential mobility and diffusibility of AMELX and AMBN in mandibular bone. This last point is an important consideration when ascribing growth factor-like or cell signaling attributes to AMELX and AMBN.

## Materials and Methods

### Animals and Tissue Sampling

The experimental animal protocol was reviewed and approved by the French Ministry of Agriculture for care and use of laboratory animals (B2 231010EA). All experiments were performed in accordance with the French National Consultative Bioethics Committee for Health and Life Science, following the ethical guidelines for animal care. All procedures related to AMELX KO and their Wild-Type (WT) littermates were reviewed and approved by The Institutional Animal Care and Use Committee (IACUC) of the University of Pennsylvania (Protocol # 803067, “Enamel Mineral Formation during Murine Odontogenesis”).

WT Swiss male mice (Janvier, St Berthevin, France) at 1, 8 and 15 weeks of age and 1 and 8 week old AMELX KO mice [26] were obtained.

**Figure 1:**
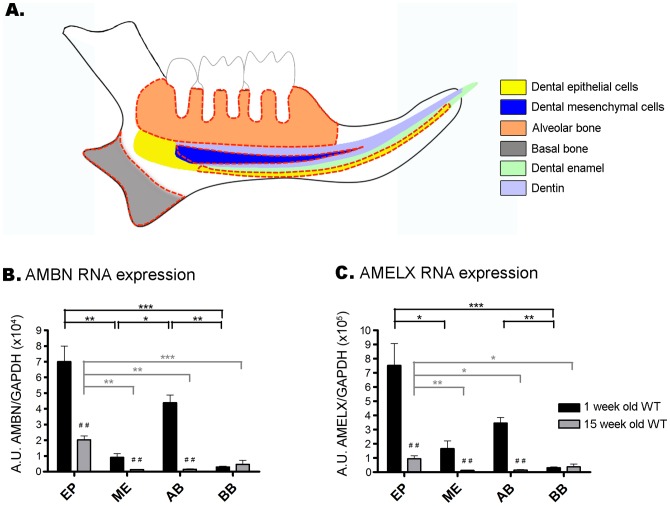
AMBN and AMELX mRNA expression in mandible tissues from 1 and 15 week old WT mice. **A.** Illustration of the harvested zones in mandible. Alveolar bone (AB), basal bone (BB), dental epithelial cells (EP) and mesenchymal cells (ME) were microdissected under a stereomicroscope (red dotted lines). Soft tissues, erupted (root and crown) and unerupted (dental germ) molars were carefully removed. AB is composed of bone tissue surrounding extracted molars and, when molars are not erupted, of bone cavity surrounding molar tooth germs (Orange zone). BB is collected from the mandible angular process (Grey zone). Harvested zones of EP (Yellow zone) and ME (Dark blue zone) from the continuously erupting incisor are delimited by red dotted lines. **B–C.** Quantitative PCR reactions were performed on EP, ME, AB and BB tissues from 1 week and 15 week old WT mice with AMBN or AMELX primers (see primer sets in Material and Methods). mRNA expression levels were normalized against expression of the housekeeping gene GAPDH. Significant differences for each tissue between different stages (MW test) and between tissues at the same stage (KW test) are indicated on the graphs. Note that the apparent reduction in AMBN and AMELX mRNA expression in enamel epithelium in older mice might be due to increased proportion of maturation and post maturation stage ameloblasts in the enamel epithelium harvested from older mice.

As detailed in Fig. 1, alveolar and basal mandible bones and dental epithelial and mesenchymal cells from 1 and 15 week old WT mice were microdissected under a stereomicroscope (Leica MZ FLIII, Leica Microscopy Systems, Ltd., Heerbrugg, Switzerland). The molar alveolar bone (AB) was harvested after removal of the mandibular soft tissues and molars. The exfoliation of forming tooth germs and formed teeth was performed by careful observation under stereomicroscope and using miniaturized Gracey curette. Basal bone (BB) was collected from the mandible angular process in order to exclude both dental and cartilage tissues. The harvested bone tissues (AB and BB) were carefully rinsed in 1× Dulbecco's phosphate-buffered saline (DPBS, Invitrogen, Carlsbad, CA, USA) to avoid soft tissue contamination. Dental epithelial cells (EP) and dental mesenchymal cells (ME) were dissected as previously described [27]. Briefly, continuously erupting incisors were carefully extracted and the dental epithelial cells (EP) were harvested by stripping the entire epithelium tissue off the incisor buccal surface, excluding the cervical loop to prevent contamination by dental stem cells (Fig. 1). Consequently, dental epithelial cells isolated from incisors of 1 week old mice were mainly composed of enamel-forming cells harvested from the secretion and maturation stages. Dental epithelial cells were similarly harvested from 15 week old mice but samples obtained from these older animals contained proportionally less tissue from the secretory stage as the proportion of epithelial cells present in the maturation stage and post maturation stage (*i.e.* protective reduced enamel epithelium) increases with age. Incisors were then cleaned and fractured to collect dental mesenchymal cells (ME) from dental pulp. Finally, a panel of non-mineralized tissues (eye, tongue, liver, heart, lung, kidney, colon, ovary, testicle and striated muscle) were dissected from 15 week old WT mice and washed in 1× DPBS. All quantitative experiments were performed in triplicate with at least three animals for each time point. After dissection, tissues were immediately frozen and stored at −80°C.

### RNA Extraction and RT-qPCR

Tissues were mechanically ground to a fine powder in liquid nitrogen and RNA was extracted using the TriReagent kit (Euromedex, Souffelweyersheim, France), following the manufacturer's instructions. In brief, total RNA was precipitated with isopropanol and centrifuged at 12,000 g at 4°C. Then, the RNA pellet was washed with 75% ethanol and resuspended in RNase-free water. The concentration and purity of total RNA in each sample were determined by the A260/A280 ratio. The integrity of RNA was confirmed by electrophoresis on an agarose ethidium bromide gel. One microgram of total RNA from each sample was reverse transcribed into cDNA using 200 units of Superscript II (Invitrogen) and 250 ng of random primers according the manufacturer's instructions. Real-time PCR reactions were performed using a MiniOpticon Real-Time PCR Detection System (Bio-Rad Laboratory, Hercules, CA, USA). According to manufacturer's recommendations, a 15 µl volume containing 7.5 µl of IQ SYBR Green Supermix (Bio-Rad Laboratory), 50 ng of cDNA as template and 0.3 µM of the appropriate primer-pairs (Eurogentec, Liège, Belgium) was used. The final mixture was subjected to PCR under the following conditions: denatured at 98°C for 10 s, followed by 40 amplification cycles (98°C for 10 s, 60°C for 30 s, and 72°C for 20 s). AMBN and AMELX PCR amplified products were resolved on a 2% agarose gel and their specificity was confirmed by sequencing (GATCBiotech, Mulhouse, France). mRNA levels of genes of interest were normalized against gene expression of the housekeeping gene GAPDH. The following specific primers sets were used: AMBN (F5′-agctgatagcaccagatgag-3′/R5′-tggcctatggaactctgttc-3′), AMELX F5′-aagcatccctgagcttcaga-3′/R5′-actggcatcattggttgctg-3′ and GAPDH (F5′-gaccccttcattgacctcaacatc-3′/R5′-aagttgtcatggatgaccttggcc-3′).

### In situ hybridization using Amelogenin oligonucleotidE probes

*In situ* hybridization using digoxygenin (DIG)-labeled oligonucleotidic probes (sequence - gaggtggtaggggcatagcaaaa - Exiqon, Vedbaek, Denmark) was performed as previously described [28]. Briefly, sections were deparaffinized using Clearene solvent (Leica Microsystems, Nanterre, France) and rehydrated through ethanol solutions diluted in *in situ* hybridization buffer (0.1% Diethyl pyrocarbonate (DEPC, Sigma-Aldrich Co., St. Louis, MO, USA) in DPBS). After three washes in *in situ* hybridization buffer, sections were subjected to proteinase-K treatment in a humid chamber. After washing with saline-sodium citrate (SSC) buffers, sections were blocked using DIG blocking reagent in buffer containing 10% heat-inactivated sheep serum, and hybridization detected using alkaline phosphatase-conjugated anti-DIG, in conjunction with 4-nitro-blue tetrazolium (NBT) and 5-bromo-4-chloro-3′-Indolylphosphate (BCIP) substrate (Roche, Mannheim, Germany) together with 2 mM Levamisole (Dako, Glostrup, Denmark). Sections were mounted with mounting resin (Eukit, Agar Scientific, Freiburg, Germany) and observed using a Leica DMRB microscope (Leica Microscopy Systems).

### Immunohistochemistry (IHC)

#### Tissue Preparation

After dissection, mandibles were fixed for 24 h in 4% paraformaldehyde (PFA, Sigma-Aldrich Co.) in 1× DPBS (Invitrogen) and washed with 1× DPBS for 1 h. Samples isolated from mice older than one week (*i.e.* 8 and 15 week old mice) were then decalcified in buffered 4% ethylene diaminetetraacetic acid (EDTA) solution at 4°C (under agitation from 1 up to 15 weeks). Finally, the samples were dehydrated, cleared in xylene and embedded in paraffin. Then, 8–10 µm sections were cut.

#### Immunohistoperoxidase

After deparaffinization and rehydration, endogenous peroxidase was inactivated by treatment with 3% H_2_O_2_ (Merck, Darmstadt, Germany) in 1× DPBS for 15 min. Sections from 1 and 8 week old WT and AMELX KO mice were then rinsed in 1× DPBS and blocked overnight at 4°C with ready-to-use (2.5%) normal horse blocking reagent (ImmPRESS reagent kit, Vector Laboratories, Burlingame, CA, USA). Sections were then probed with primary anti-AMBN antibody (1/100) (sc 50534 (M-300), Santa Cruz Biotechnology - rabbit polyclonal IgG to AMBN - Immunogen: amino acids 108–407 mapping at the C-terminus of AMBN mouse origin - Reacts with mouse) and anti-AMELX antibody (1/100) (ab 59705, Abcam, Cambridge, MA, USA - rabbit polyclonal IgG to AMELX - Immunogen: purified full length native protein of AMELX cow origin – Reacts with mouse, rat and cow) for 1 h at room temperature. Tissue sections were washed three times with 1× DPBS for 5 min and incubated for 30 min with ImmPRESS reagent (ImmPRESS reagent anti-rabbit Ig Kit, Vector Laboratories, Burlingame, CA, USA). After three washes in DPBS 1X, immuno cross-reactivity was visualized using diaminobenzidine peroxidase substrate (Novared, Vector Laboratories). Sections were dehydrated and mounted in resin (Eukit, Agar Scientific, Freiburg, Germany). Sections with no primary antibodies were used as negative controls.

#### Immunohistofluorescence

After deparaffinization and rehydration, mandible tissue sections from 1 week old WT and AMELX KO mice were permeabilized for 10 min in 1% Triton X-100 (Thermo Fisher Scientific Inc, Waltham, MA, USA), then rinsed with 1× DPBS 3 times for 5 min each under agitation. Nonspecific binding sites were blocked by 30 min incubation in 1% bovine serum albumin (BSA, Euromedex, Mundolsheim, France) diluted in DPBS. Sections were incubated overnight at 4°C with primary anti-AMBN antibody (1/200) (sc 50534 (M-300), Santa Cruz Biotechnology) and anti-AMELX antibody (1/200) (ab 59705, Abcam). After washes with 1× DPBS, Alexa Fluor 594 or 488 Goat Anti-Rabbit IgG antibodies (1/500) (Thermo Fisher Scientific Inc) were applied for 1 h at room temperature followed by DAPI nuclear staining (1/100,000) (Sigma-Aldrich Co.). Sections were mounted using an aqueous mounting medium (Fluoprep, Biomérieux, France).

### Western Blot Analysis

To extract total proteins, mashed tissues from 1 and 15 week old WT mice were homogenized in 300 µL of commercial protein extraction reagent T-PER (Thermo Fisher Scientific Inc) which contains the detergent bicine. Samples were sonicated and tissue debris, removed by centrifugation for 5 min at 10,000 rpm. Supernatants were recovered and protein concentration determined using the BCA protein assay (Thermo Fisher Scientific Inc). Samples were prepared for SDS PAGE by adding 100 µL of Laemmli sample loading buffer containing 10% DTT (Bio-Rad Laboratory). Samples were heated for 15 min at 70°C and stored at −20°C. Samples were loaded at 10 µg protein per well on 12% polyacrylamide gels (Prosieve 50, Lonza, Rockland, USA).

Extraction of proteins present in the fluid phase of the tissues was carried out as previously described [29]. Briefly, dissected samples were immersed in 30 µL of TRIS solution (50 mM, pH 7.4), crushed and centrifuged for 4 min to 20 000 g. Then, the supernatant was recovered, diluted in 1∶1 Laemmli buffer (v/v) and stored at −80°C. Protein concentration in extracts was determined using a ready-to-use assay compatible with samples containing Laemmli SDS sample buffer, according to the manufacturer's instructions (Pierce 660 nm Assay, Thermo Fisher Scientific Inc). Protein samples were thawed and heated 5 min at 100°C, before loading at 10 µg protein per wells on a 10% polyacrylamide gel.

Electrophoresis was carried out for 75–90 min at 180–200 V prior to transfer onto PDVF membranes (Thermo Fisher Scientific Inc) for 45–60 min at 100 Volts. Membranes were stained to check the efficiency of the electro-transfer (Novagen RedAlert, EMD Millipore, Billerica, MA, USA). Membranes were blocked overnight using 5% milk powder in 1× DPBS, then washed and incubated 1–2 h with anti-AMBN antibody (1/400) (sc 50534 (M-300), Santa Cruz Biotechnology) or anti-AMELX antibody (1/400) (sc 32892 (FL-191), Santa Cruz Biotechnology - rabbit polyclonal IgG to AMELX - Immunogen: amino acids 1–191 representing full length AMELX isoform of human origin - Reacts with mouse, rat and human). After rinsing, membranes were incubated with a horseradish peroxidase (HRP)-conjugated goat anti-rabbit IgG antibody (Sigma-Aldrich Co.) at 1/2,000 dilution. Finally, immunocross-reactivity was visualized by chemiluminescence using HRP substrate (Luminata Crescendo, Millipore Co., Billerica, USA) and a LAS 4000 Bioimager (ImageQuant, Uppsala, Sweden).

### Statistical analysis

Data are presented with values expressed as the mean ± standard error of the mean (m ± SEM). Statistical analyses of the Q-PCR data were performed using Prism 5 statistical software (GraphPad Software Inc., San Diego, CA). Non-parametric two-tailed tests were used to compare EMP expression; Mann-Whitney (MW) test to investigate the EMP expression in each tissue between different stages (1 week vs 15 week old mice) and Kruskal-Wallis (KW) test to compare the difference between tissues (EP, ME, AB, BB) at one specific stage. The overall risk was fixed at p<0.05 (*p<0.05, **p<0.01 ***p<0.001 (KW test), ^#^p<0.05, ^# #^p<0.01 ^# # #^p<0.001 (MW test), NS; not significant).

## Results

### AMBN and AMELX RNA expression in mineralized and non-mineralized tissues

Screening of AMBN and AMELX gene expression in murine tissues revealed the expression of transcripts coding for matrix enamel proteins in tooth, bone and soft tissues (Fig. 1 and Table 1). Quantitative analysis of AMBN and AMELX mRNA expression in the mandible tissues from 1 week old and 15 week old mice was performed using RT-qPCR (Fig. 1). In mice of both age groups AMBN and AMELX mRNA expression was not only detected in dental epithelial cells (EP) and mesenchymal cells (ME) but also in alveolar bone (AB) and basal bone (BB) (Fig. 1B-C). Overall, AMBN and AMELX genes had similar expression patterns, with RNA levels highly dependent on the investigated tissue and the age of the donor mouse. In 1 week old WT mice, AMBN and AMELX mRNA levels were significantly enhanced in EP when compared to ME and BB (p<0.05–0.001), whereas mRNA levels in EP and in AB remained comparable (NS). In 15 week old mice, however, AMBN and AMELX mRNA levels were significantly higher in EP when compared to the three other tissues (p<0.05-0.001). Finally, when compared to 1 week old mice, AMBN and AMELX mRNA expression was significantly decreased in 15 week old mice in all investigated tissues (p<0.01), except in BB where mRNA levels remained comparable (NS). A series of non-mineralized extra-dental tissues from adult WT mice (15 week old) were also screened for AMBN and AMELX mRNA expression. Soft tissues were dissected and RNA was prepared for RT-PCR. Dental epithelial cells (EP) harvested from incisors were used as positive control tissue. RT-PCR results for these tissues are listed in Table 1. It is important to note that, when detected in soft tissues, AMBN and AMELX showed low mRNA levels when compared to mandibular tissues. Among the 10 soft tissues screened, only liver, lung and striated muscle showed no AMBN and AMELX mRNA expression; identifying these tissues as reliable negative controls for expression of these two EMP genes. Based on these results, striated muscle tissue was subsequently used as negative control tissue for both AMELX and AMBN gene expression in *in situ* hybridization and immunohistochemistry (IHC) performed on jaws.

**Table 1:** Ameloblastin and amelogenin mRNA expression in murine tissues.

Tissues	AMBN mRNA	AMELX mRNA
Dental epithelial cells	++	++
Dental mesenchymal cells	++	++
Mandibular alveolar bone	++	++
Mandibular basal bone	++	++
Eye	+	+
Tongue	+	+/−
Testicle	+	+/−
Heart	+/−	+/−
Colon	+/−	+/−
Ovary	-	+
Kidney	-	+/−
Liver	-	-
Lung	-	-
Striated muscle	-	-

Tissues were dissected from 15 week old WT mice (n = 6 with n = 3 females and n = 3 males) and subjected to RT-PCR (see Materials and methods). Resulting products were resolved on a 2% agarose gel. AMBN positive tissues show one amplicon band at 287 bp and AMELX positive tissues show at least one of the three amplicon bands at 415 bp, 373 bp and 303 bp corresponding to transcript variants encoding different isoforms of AMELX described in the literature; at 415 bp (M217 [67]–M194 [68]), 373 bp (M203 [69]–M180 [70]) and 303 bp (M179 [69]–M156 [70]). +, PCR products were repeatedly obtained; +/−, not all samples were positive; -, no PCR products were visible. The overall high signal in mRNA levels (++) in mandibular mineralized tissues led to perform additional RT-qPCR analyses.

### Spatiotemporal localization of AMBN and AMELX

**Figure 2:**
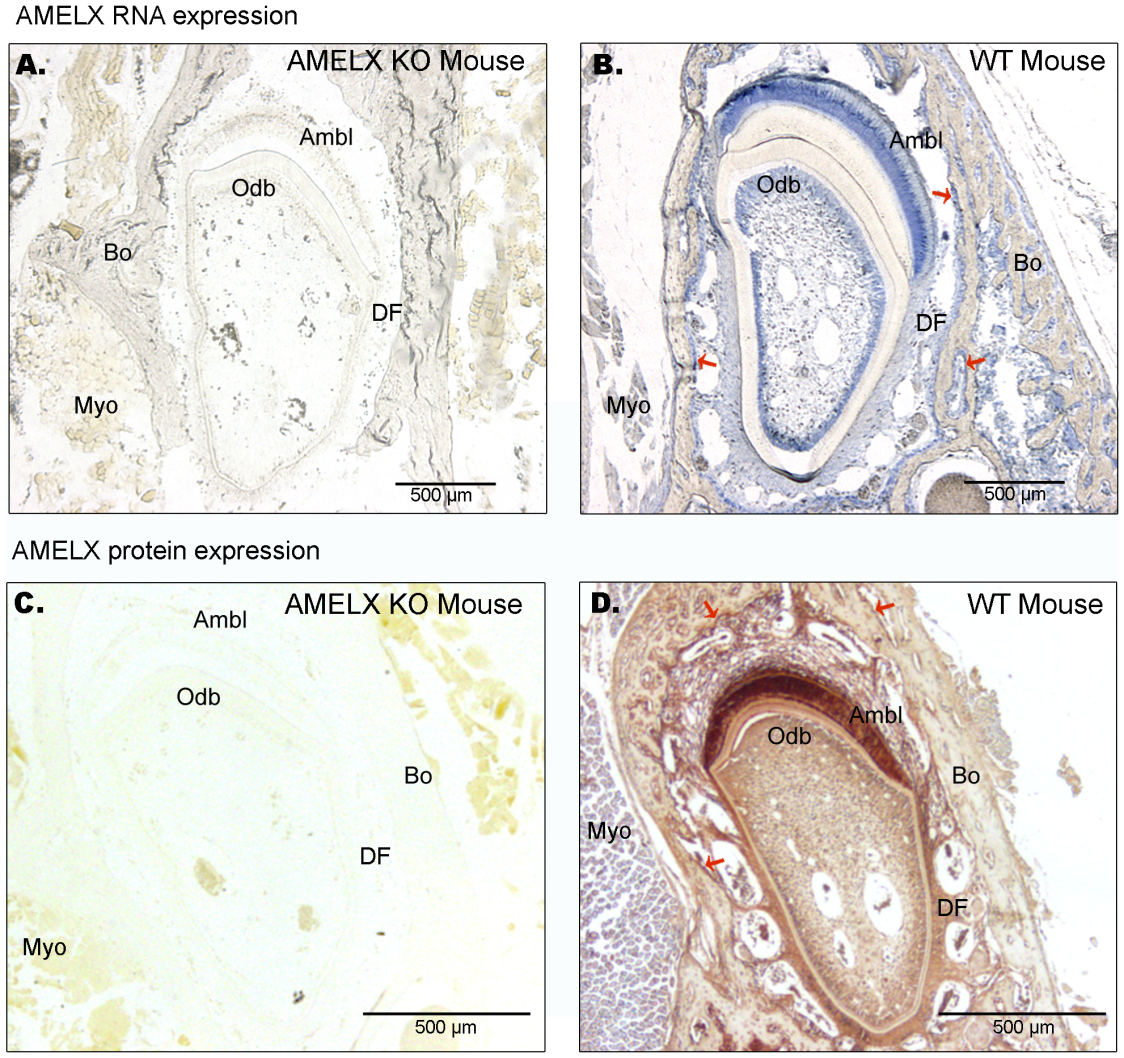
AMELX mRNA and protein distribution in mandible from 8 week old WT and AMELX KO mice. **A-B.** *In situ* hybridization was performed using AMELX oligonucleotidic probes. **B.** WT mouse shows strong AMELX mRNA level in ameloblasts (Ambl) and odontoblasts (Odb). AMELX mRNA is also detected in bone-lining cells (red arrows) and, with an apparent lower level, in dental follicle (DF) area. **C–D.** Immunohistodetection was performed using anti-AMELX antibody. **D.** AMELX protein expression in WT mouse was detected in ameloblasts, odontoblasts and bone-lining cells (red arrows). Strong and diffuse protein signal was also observed in dental follicle. **B–D.** No AMELX RNA and protein signal is detected in striated muscle (Myo), a negative control tissue (see Table 1). **A–C.** Neither AMELX mRNA nor protein expression is detected in AMELX KO mice.

**Figure 3:**
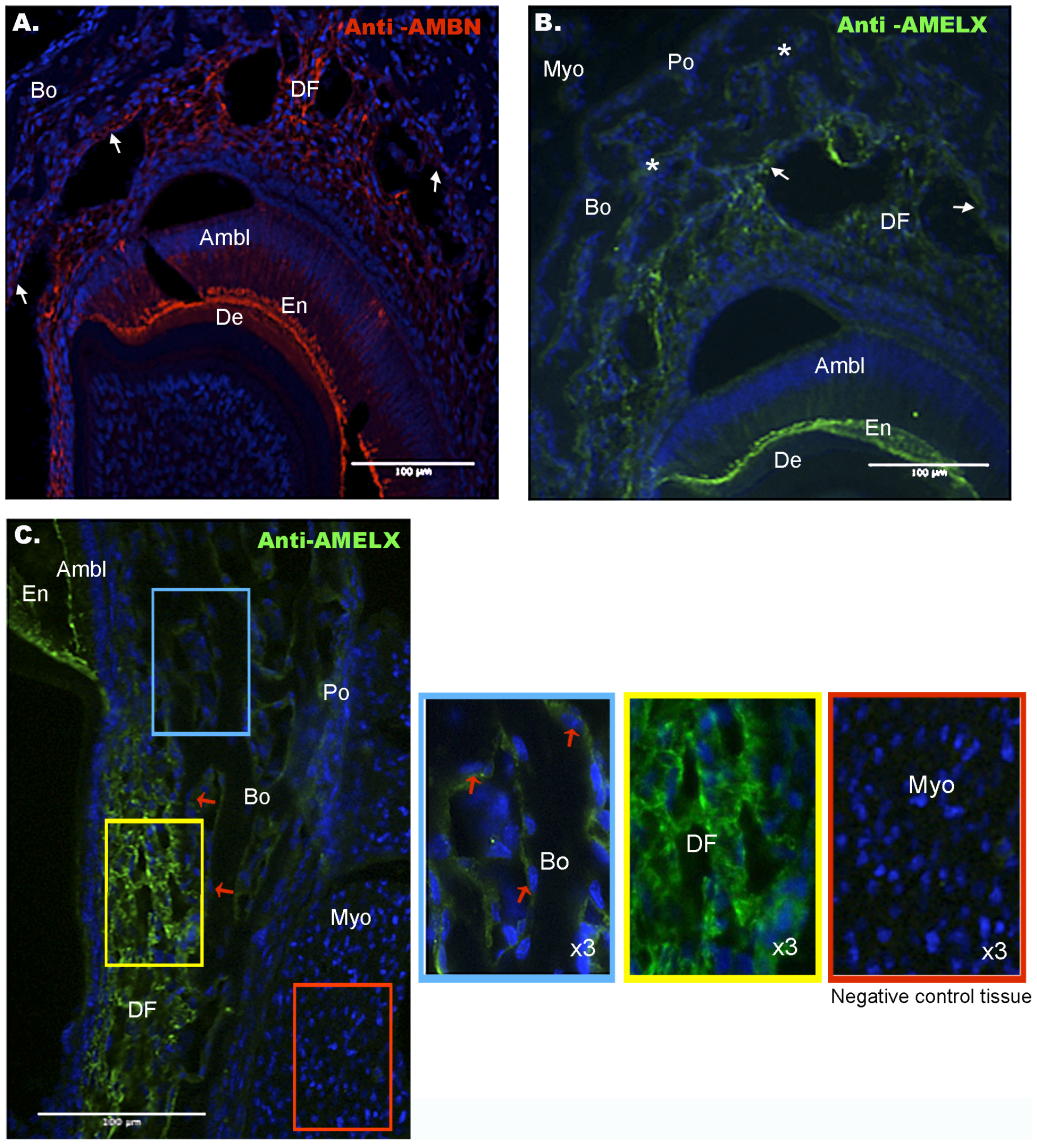
AMBN and AMELX protein expression in 1 week old WT mice. **A.** AMBN protein (red signal) is strongly expressed in enamel (En) and in dental follicle (DF) and is detected in cells lining alveolar bone (white arrows). **B.** On serial sections, AMELX protein (green signal) shows similar localization pattern with expression in enamel, dental follicle and bone-lining cells (white arrows). In addition, a diffuse AMELX signal is also detected in periosteum (Po) and bone (Bo) (in particular in matrix of trabebular spaces (white asterisks)). No protein expression is detected in striated muscle (Myo), a negative control tissue. **C.** Higher magnification of AMELX protein expression shows AMELX-positive osteoblastic cells lining bone trabeculae (red arrows) (**Blue box**), strong expression in dental follicle area (**Yellow box**). Higher magnification shows no signal in striated muscle (**Orange box**). Ambl  =  ameloblast, Bo  =  bone, De  =  dentin, DF  =  dental follicle, En  =  enamel, Myo  =  striated muscle and Po  =  periosteum.

Based on RT-PCR data (Fig. 1 and Table 1) and a range of primary antibody dilutions tested against sections of mandible containing EMPs-expressing ameloblasts (positive control tissue), jaw bones and striated muscle (negative biological control, as evidenced by RT-PCR described above - Table 1), we determined two dilution thresholds for detection of EMPs; one for enamel and one for bone (Fig. S1). Additionally, to avoid non-specific staining we used a “triple negative-control system” including: 1- sections of dental epithelium where primary antibody was omitted (data not shown); 2- mandibular sections from WT mice including striated muscle, and, for anti-AMELX IHC, 3- mandibular sections from AMELX KO mice. AMELX labelling in AMELX KO tissues and in striated muscle (Myo) from WT mice was negative (Fig. 2, Fig. 3, Fig. S1 and Fig. S2).

In contrast to the age-dependent differential gene expression revealed by RT-qPCR (Fig. 1), the distribution pattern of AMELX and AMBN was identical at all studied ages in the mandibular tissues surrounding the incisor. *In situ* hybridization in mandible confirmed AMELX mRNA expression in ameloblasts (positive control tissue) and, with lower intensity, in the alveolar bone area; where bone-lining cells showed AMELX mRNA expression (Fig. 2B). AMELX protein was visualized using IHC and showed similar but more diffuse localization pattern when compared to AMELX mRNA distribution (Fig. 2D). No RNA nor protein signal was observed in mandible tissues from 8 week old AMELX KO mice (Fig. 2A–C).

AMBN and AMELX protein distribution in manbibular sections from 1 week old WT and AMELX KO mice was further investigated using immunohistofluorescence staining (Fig. 3-Fig. S2). Besides their presence within enamel matrix, both AMBN and AMELX proteins were detected in mandibular bone-lining cells (Fig. 3.A–B, white arrows, Fig. 3C, inset blue box) and in dental follicle (DF) (Fig. 3A-B-C, inset yellow box) in WT mice. Additionally, a slight diffuse expression of AMELX was detected in the periosteum (Po) and bone (Bo) areas (Fig. 3B and Fig. 3C).

### Western blot detection of AMELX and AMBN in mandibular bone

**Figure 4:**
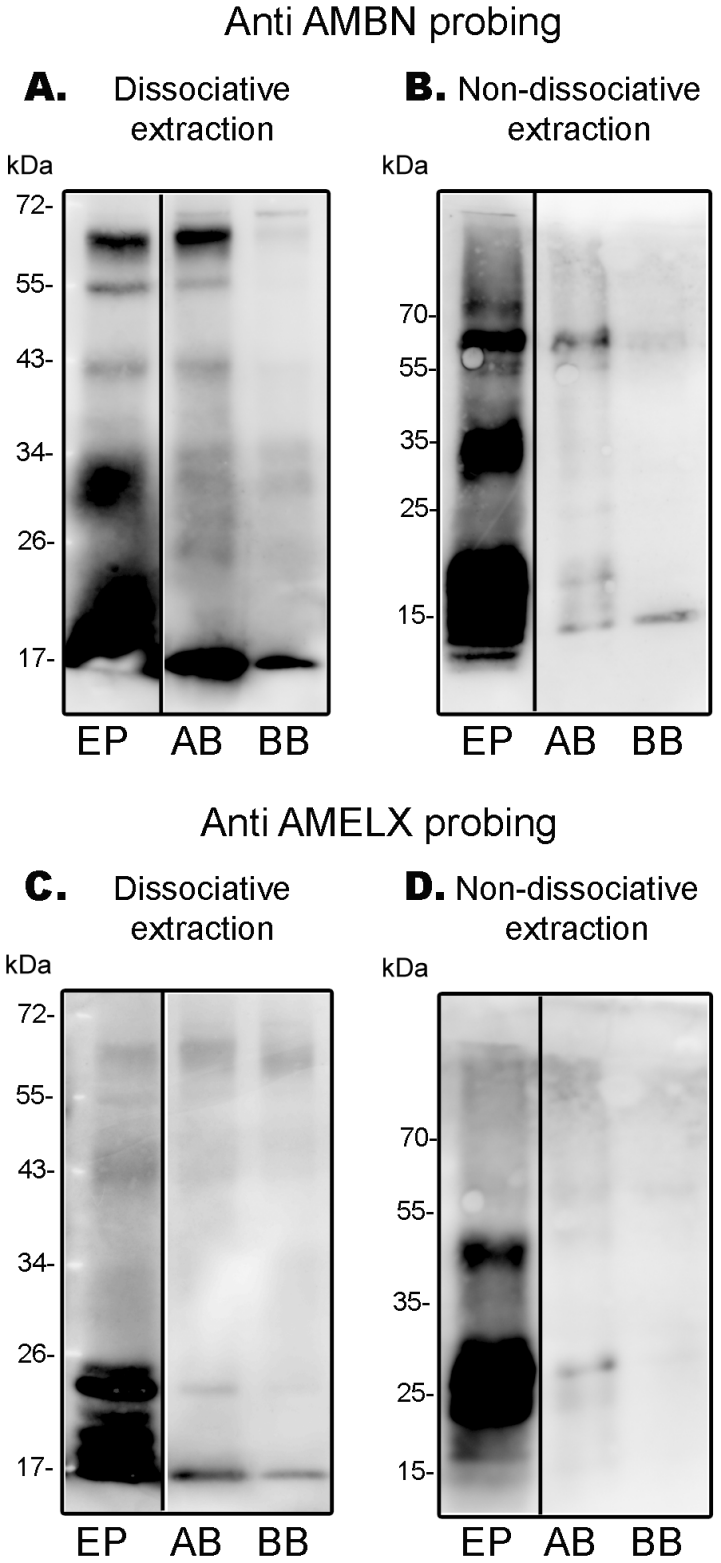
Western blot of AMBN and AMELX in 15 week old WT mice. Proteins were extracted from dental epithelial cells (EP) (positive control), alveolar bone (AB), basal bone (BB) under dissociative and non-dissociative conditions. Proteins were loaded at 10 µg per lane and blots probed with anti-AMBN and anti-AMELX antibodies. **A.** Anti-AMBN probing of dissociative extracts show cross reactive species with molecular weights ranging from 20–67 kDa for AMBN (the 67 kDa band corresponds to nascent amelobastin). 67 kDa AMBN is present at similar relative amounts in the EP and AB extracts but far less readily detectable in BB. **B.** A similar situation exists for non-dissociative extracts. **C.** Anti-AMELX probing of dissociative extracts show cross reactive species at 26 kDa and below in EP samples (higher molecular weight staining may be due to AMELX aggregation). Feint cross reactivity at ∼25 kDa is visible in AB samples with even less been detected in BB; the relative amount of AMELX present in bone samples is far less than that seen in EP samples. **D.** A similar situation exists for non-dissociative extracts; AMELX is detectable in AB but it is present in the extract in much lower amounts compared to EP extracts.

To analyze the protein expression profile of EMPs in alveolar (AB) and basal bones (BB), we first studied total protein extracts from 15 week old mice using a detergent-based dissociative extraction procedure. Dental epithelium cells (EP) were used as positive controls. Extracted proteins were separated by SDS PAGE (10 µg total protein per well) and transferred to membranes for western blotting. Anti-AMBN immunostaining of the western-blot (Fig. 4A) revealed the presence of the nascent AMBN molecule at 67 kDa and a range of lower molecular weight processing products in dissociative extracts of dental epithelial cells. A similar molecular weight profile was reported for ameloblastin extracted from rat incisor enamel organ [30]. The nascent 67 kDa ameloblastin was also detected in dissociative extracts of alveolar bone (AB) with lower molecular weight processing products. AMBN immuno-cross reactivity in basal bone (BB) was much weaker and was near the limit of detection. Anti-AMBN immunostaining of blots of non-dissociative extracts of EP, AB and BB (Fig. 4B) revealed a spectrum of AMBN staining similar to the dissociative extracts. Anti-AMELX immune staining of dissociative extracts showed the presence of multiple AMELX proteins migrating below 26 kDa in the enamel epithelium (Fig. 4C). The additional lower weight AMELX components may be AMELX processing products from enamel matrix present as a contaminant in dental epithelium cell extracts. The identity of the stained bands migrating at 43 kDa and above is unclear though amelogenin is known to form high molecular weight complexes that are stable during SDS PAGE [13], [31]. Dissociative extracts of AB and BB exhibited a single band of amelogenin cross-reactivity at 26 kDa though at levels near the limits of detection. Anti-AMELX immune staining of non-dissociative extracts showed the presence of AMELX as a doublet at around 26 kDa in enamel epithelium while alveolar bone exhibited a single band of amelogenin cross-reactivity at 26 kDa. A similar band was present in the basal bone extract but at such low levels that it was only visible after digitally enhancing the image (not shown).

## Discussion

To the best of our knowledge, no previous studies have quantified gene expression levels and examined the solubility states of endogenously expressed EMPs in mandible bones using dental epithelial cells as a reference in rodents. Using a similar strategy, we previously demonstrated the regulation of EMPs gene expression by nuclear effectors *in vivo* in dental [11], [27], [32] and bone tissues [33], [34]. Pioneer reports described AMELX [12], [35] and AMBN [36] in non-enamel mineralized tissues. Here we confirm and extend these data; dental epithelial cells and alveolar bone displayed higher levels of AMELX and AMBN mRNA expression when compared to basal bone and dental mesenchymal cells from post-natal mice (one week old).

By screening AMELX and AMBN gene expression in 10 soft tissues, we were able to identify the lung, liver and striated muscle as negative control tissues for *in situ* studies. In line with previous studies [15], [16], repeated or occasional mRNA expression of AMBN and AMELX was detected in several soft tissues but at low level when compared to mandibular tissues. This occasional expression could result from endogenous oscillations of EMPs gene expression orchestrated by clock genes; both AMELX and enamelin being downstream targets of the “clock genes” family of transcription factors [37], [38]. Alternatively, these variations may result from the existence of EMPs-positive circulating cells (*e.g.* macrophages, megakaryocytes and some hematopoietic stem cells [15]).

Our RT-qPCR and western-blot results show that AMELX and AMBN expression is higher in alveolar bone compared to basal bone. This finding raises the possibility that AMELX and AMBN are locally associated with high bone turn-over; this being a classic characteristic of alveolar bone [39]. Indeed, AMELX [25] and AMBN [40] have been shown to impact on osteoblast and osteoclast differentiation and activity. Consistently and in line with previous studies [40], aging was associated with decreased gene expression of EMPs in jaw bones. Recently, increased expression of AMBN was also shown to be associated with mechanical bone stimulation [41] and healing [42]. Interestingly, MSX2, a regulator of bone homeostasis [33] represses AMELX transcription by targeting the AMELX promoter [43] and both AMELX and AMBN inhibit Msx2 expression [44], [45]. EMPs-Msx2 reciprocal cross-talk may thus play a part in localized jaw modeling as supported by Msx2 and AMBN null-mutants [34], [44] and our preliminary biomechanical assay (Fig. S3). Indeed, 72 h after altering occlusion, we observed a significant increase in AMBN and AMELX mRNA expression associated with decrease in Msx2 gene expression.

**Figure 5:**
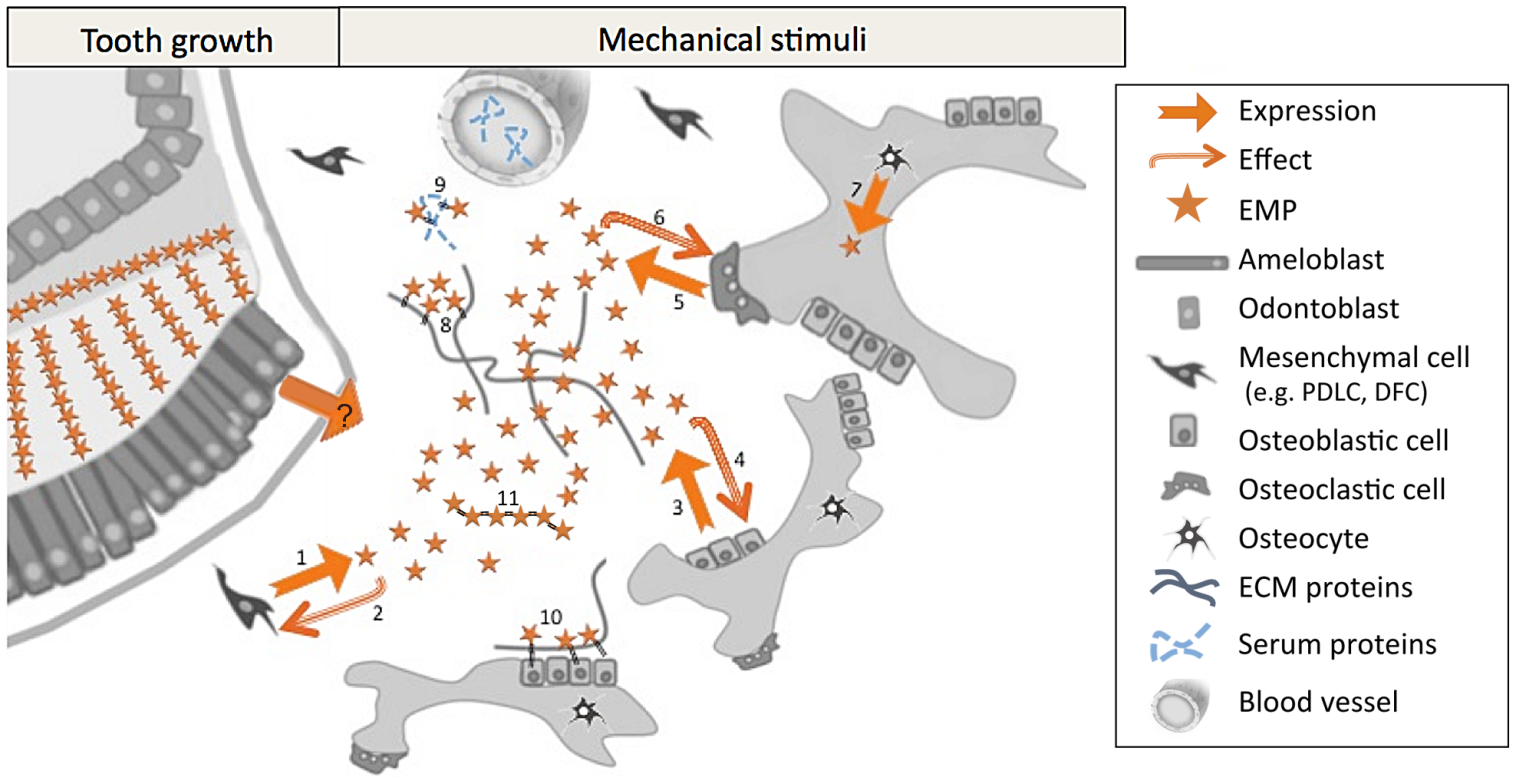
Proposed model for EMPs expression and signaling in extra-dental tissues. This model is based on the presently described RNA and protein patterns and published data. EMP-based autocrine and paracrine cell-cell communications would participate to distinct physiopathological events. They may play a role during tooth growth and alveolar bone modeling processes. In adults, EMPs would be involved in alveolar bone responses to mechanical stimuli. Supramolecular structures generated by self-assembly of EMPs might also intervene in these processes. **Bibliographical references. 1.** Haze, 2009 [8]; Tamburstuen, 2011 [10], **2.** Zeichner-David, 2006 [57]; Fukae, 2006 [58]; Matsuzawa, 2009 [59]; Tambursten, 2010 [42]; Kakegawa, 2010 [60]; Zhang, 2011 [54]; Kitagawa, 2011 [53]; Kunimatsu, 2011 [61]; Izumikawa, 2012 [62]; Amin, 2013 [63], **3.** Spahr, 2006 [36]; Haze, 2009 [8]; Iizuka, 2011 [18]; Tamburstuen, 2011 [10]; Atsawasuwan, 2013 [40], **4.** Matsuzawa, 2009 [59]; Iizuka, 2011 [18]; Tamburstuen, 2010 [42]; Amin, 2013 [64]; Atsawasuwan, 2013 [24], **5.** Haze, 2009 [8]; Tamburstuen, 2011 [10], **6.** Tamburstuen, 2010 [42]; Lu, 2013 [19], **7.** Haze, 2009 [8], [20], **8.** Saito, 2008; Wang, 2005 [48], **9.** Wang, 2005 [48], **10.** Beleyer, 2010 [47], **11.** Fincham, 1994 [65], Wald, 2013 [66].

Protein occupies 20–30% of the secretory stage enamel matrix by volume and of this protein >90% is derived from the AMELX gene whereas AMBN is present in the matrix at far lower concentrations. AMELX is highly aggregative under physiological conditions and acts as a structural scaffold during enamel development. Nascent AMBN is solubilized in the developing enamel matrix and is a mobile species. In contrast, AMBN processing products are more aggregative [29]. Identifying solubilized and diffusible proteins in tissues using histology-based techniques may be compromised by the fact that otherwise soluble factors become immobilized by chemical fixation. Here AMELX and AMBN proteins were detectable in the microenvironment surrounding EMPs-producing (RNA-positive) cells. In addition, high protein levels detected in the dental follicle suggest that dental and bone cells may secrete these peptides toward this adjacent tissue. If EMPs function in some capacity as signaling molecules [12] then endogenously secreted EMPs would require a certain degree of solubility in the extracellular environment in order for them to diffuse and interact with nearby cells (paracrine signaling) or receptors on the secreting cell itself (autocrine signaling) (Fig. 5). To test this hypothesis, we extracted dental epithelial cells (EP), alveolar bone and basal bone under dissociative and non-dissociative conditions and subjected equal amounts of total protein extracted to western blot probing for AMBN and AMELX. We provide evidence that AMBN, particularly the nascent 67 kDa molecule, is readily detectable on blots of dissociative extracts of alveolar bone; and it is present in these extracts at a similar ratio relative to other components as found in EP. The ratio of freely soluble 67 kDa AMBN in the non-dissociative extracts is also comparable to the ratio found in EP; *i.e.* to some degree, AMBN is present in alveolar bone in a solubilized state; or at least it is not tightly associated with the bone matrix. AMELX was also detectable in blots of non-dissociative extracts of alveolar bone but it comprised a far smaller ratio of the dissociatively extracted proteins when compared to EP. Likewise, AMELX was detectable in non-dissociative extracts of alveolar bone but again at ratios far lower than the ratio of AMELX in EP.

We were unable to detect EMPs in serum (data not shown), which suggests that, contrary to osteocalcin that behaves as hormonal signal in general metabolism [46], EMPs might not play a role in long-range endocrine signaling. Some matrix ligands, omnipresent in mesenchymal extracellular compartments, could perhaps entrap these secreted EMPs and limit their diffusion. Indeed, fibronectin [47], heparan sulfate [20] and biglycan [48] bind AMELX or AMBN *in vitro*. However, the inability to detect EMPs in serum may simply be due to dilution of the bone-derived EMP peptides once they enter the blood stream.

Autocrine and paracrine activities of growth factors control local bone modeling and remodeling. Solubilized EMPs may behave in the same way (Fig. 5). Indeed, distinct dysmorphologies characterize bones in which AMELX [16], [49] or AMBN [24], [50] expression are genetically modified, even though calcium and phosphorus content and the degree of mineralization are normal [51], [52]. Disturbed EMP expression patterns are associated with alveolar and jaw bone malformations [33], [34]. These findings and the present data suggest that solubilized EMPs may play a role in controlling cell fate through short-distance paracrine and autocrine functions and thus control local bone morphology *via* signals previously evidenced for AMBN [19], [53], [54] and AMELX [55], [56]. We thus propose a model in which EMPs play a role in mandibular physiology (Fig. 5).

## Conclusion

Based on our findings on the solubilized state and the localization of AMELX and AMBN in mandibular bones, we hypothesize that these EMPs play a dual role: one as components of a structural extracellular matrix in developing enamel and second as growth factor-like molecules in bones responsible for supporting the teeth in the mandible.

## Supporting Information

Figure S1 **Optimization of AMBN and AMELX immunodetection in 1 week old WT and AMELX KO mice.** **A-B**. When using a classic dilution of antibody (1/1,000), cross reactivity to AMELX and AMBN proteins is restricted to the dental epithelial cells (EP) (positive control tissue) in WT mouse, a pattern well documented in the literature. Dilutions of 1/500 show the presence of AMBN and AMELX protein expression in EP, but also in some bone stromal cells. Increased concentration of antibody (1/100 for AMBN and 1/50 for AMELX) results in stronger staining in osteoblasts, in osteocytes and in mandibular bone matrix. At dilution 1/100 (AMBN) or 1/50 (AMELX), striated muscle (negative control tissue) shows diffuse staining indicating the limit of specificity for these antibodies. At dilution 1/10, the striated muscle was clearly stained for both AMBN and AMELX, determining the non-specificity threshold. This threshold was confirmed using AMELX KO mouse as a *bona fide* control, non-specific cross reactivity signal being observed when using 1/10 antibody dilution. For detailed immunohistoperoxidase methods see File S1.
(TIF)

Figure S2 **AMELX protein expression in 1 week old WT and AMELX KO mice.** **A.** When using 1/500 antibody dilution, AMELX (green staining) is not detected in AMELX KO mice. **B.** Using the same antibody dilution, WT section show anti-AMELX cross reactivity in enamel (En) and ameloblasts (Ambl).
(TIF)

Figure S3 **Impact of mechanical stimuli on AMBN and AMELX mRNA expression in jaw.** **A.** Right maxillary molar cusps in 15 week old WT mice were ground flat to the level indicated by the red dotted line. **B.** 72 h after grinding, significant increase in mRNA levels of AMELX (x7) and AMBN (x3) is observed in treated mandible alveolar bone. Concurrently, a significant reduction in MSX2 mRNA level (x3) is observed; MSX2 being a transcriptional repressor of AMELX gene. mRNA expression of those genes are unaffected in basal bone (data not shown). mRNA levels of genes of interest are normalized against mRNA expression of the housekeeping gene RS15 (F5′-ggcttgtaggtgatggagaa-3′/R5′-cttccgcaagttcacctacc-3′). Overall probability was determined using KW test (**p<0.05). For detailed molar grinding and flattening procedures see File S1.
(TIF)

File S1 **Detailed procedures.**
(DOCX)

## References

[1] Staines KA, Macrae VE, Farquharson C (2012) The importance of the SIBLING family of proteins on skeletal mineralisation and bone remodelling. J Endocrinol 214: 241–255. 22700194 10.1530/JOE-12-0143

[2] George A, Veis A (2008) Phosphorylated proteins and control over apatite nucleation, crystal growth, and inhibition. Chem Rev 108: 4670–4693. 18831570 10.1021/cr0782729 PMC2748976

[3] Kawasaki K, Buchanan AV, Weiss KM (2009) Biomineralization in humans: making the hard choices in life. Annu Rev Genet 43: 119–142. 19659443 10.1146/annurev-genet-102108-134242

[4] Iwata T, Yamakoshi Y, Hu JC, Ishikawa I, Bartlett JD, et al (2007) Processing of ameloblastin by MMP-20. J Dent Res 86: 153–157. 17251515 10.1177/154405910708600209

[5] Bartlett JD (2013) Dental Enamel Development: Proteinases and Their Enamel Matrix Substrates. ISRN Dent 2013: 684607. 24159389 10.1155/2013/684607 PMC3789414

[6] Bosshardt DD (2008) Biological mediators and periodontal regeneration: a review of enamel matrix proteins at the cellular and molecular levels. J Clin Periodontol 35: 87–105. 18724844 10.1111/j.1600-051X.2008.01264.x

[7] Hatakeyama J, Philp D, Hatakeyama Y, Haruyama N, Shum L, et al (2006) Amelogenin-mediated regulation of osteoclastogenesis, and periodontal cell proliferation and migration. J Dent Res 85: 144–149. 16434732 10.1177/154405910608500206

[8] Haze A, Taylor AL, Haegewald S, Leiser Y, Shay B, et al (2009) Regeneration of bone and periodontal ligament induced by recombinant amelogenin after periodontitis. J Cell Mol Med 13: 1110–1124. 19228267 10.1111/j.1582-4934.2009.00700.x PMC2889159

[9] Nunez J, Sanz M, Hoz-Rodriguez L, Zeichner-David M, Arzate H (2010) Human cementoblasts express enamel-associated molecules in vitro and in vivo. J Periodontal Res 45: 809–814. 20572915 10.1111/j.1600-0765.2010.01291.x

[10] Tamburstuen MV, Reseland JE, Spahr A, Brookes SJ, Kvalheim G, et al (2011) Ameloblastin expression and putative autoregulation in mesenchymal cells suggest a role in early bone formation and repair. Bone 48: 406–413. 20854943 10.1016/j.bone.2010.09.007 PMC4469498

[11] Papagerakis P, Macdougall M, Hotton D, Bailleul-Forestier I, Oboeuf M, et al (2003) Expression of amelogenin in odontoblasts. Bone 32: 228–240. 12667550 10.1016/s8756-3282(02)00978-x

[12] Veis A, Tompkins K, Alvares K, Wei K, Wang L, et al (2000) Specific amelogenin gene splice products have signaling effects on cells in culture and in implants in vivo. J Biol Chem 275: 41263–41272. 10998415 10.1074/jbc.M002308200

[13] Landin MA, Shabestari M, Babaie E, Reseland JE, Osmundsen H (2012) Gene Expression Profiling during Murine Tooth Development. Front Genet 3: 139. 22866057 10.3389/fgene.2012.00139 PMC3408794

[14] Deutsch D, Haze-Filderman A, Blumenfeld A, Dafni L, Leiser Y, et al (2006) Amelogenin, a major structural protein in mineralizing enamel, is also expressed in soft tissues: brain and cells of the hematopoietic system. Eur J Oral Sci 114 Suppl 1: 183–189; discussion 201-182, 381 16674683 10.1111/j.1600-0722.2006.00301.x

[15] Gruenbaum-Cohen Y, Tucker AS, Haze A, Shilo D, Taylor AL, et al (2009) Amelogenin in cranio-facial development: the tooth as a model to study the role of amelogenin during embryogenesis. J Exp Zool B Mol Dev Evol 312B: 445–457. 19097165 10.1002/jez.b.21255

[16] Li Y, Yuan ZA, Aragon MA, Kulkarni AB, Gibson CW (2006) Comparison of body weight and gene expression in amelogenin null and wild-type mice. Eur J Oral Sci 114 Suppl 1: 190–193; discussion 201–192, 381 16674684 10.1111/j.1600-0722.2006.00286.x

[17] Lyngstadaas SP, Wohlfahrt JC, Brookes SJ, Paine ML, Snead ML, et al (2009) Enamel matrix proteins; old molecules for new applications. Orthod Craniofac Res 12: 243–253. 19627527 10.1111/j.1601-6343.2009.01459.x PMC2825346

[18] Iizuka S, Kudo Y, Yoshida M, Tsunematsu T, Yoshiko Y, et al (2011) Ameloblastin regulates osteogenic differentiation by inhibiting Src kinase via cross talk between integrin beta1 and CD63. Mol Cell Biol 31: 783–792. 21149578 10.1128/MCB.00912-10 PMC3028634

[19] Lu X, Ito Y, Atsawasuwan P, Dangaria S, Yan X, et al (2013) Ameloblastin modulates osteoclastogenesis through the integrin/ERK pathway. Bone 54: 157–168. 23385480 10.1016/j.bone.2013.01.041 PMC5023015

[20] Saito K, Konishi I, Nishiguchi M, Hoshino T, Fujiwara T (2008) Amelogenin binds to both heparan sulfate and bone morphogenetic protein 2 and pharmacologically suppresses the effect of noggin. Bone 43: 371–376. 18515207 10.1016/j.bone.2008.03.029

[21] Warotayanont R, Zhu D, Snead ML, Zhou Y (2008) Leucine-rich amelogenin peptide induces osteogenesis in mouse embryonic stem cells. Biochem Biophys Res Commun 367: 1–6. 18086559 10.1016/j.bbrc.2007.12.048 PMC2276612

[22] Stahl J, Nakano Y, Kim SO, Gibson CW, Le T, et al (2013) Leucine rich amelogenin peptide alters ameloblast differentiation in vivo. Matrix Biol 32: 432–442. 23747796 10.1016/j.matbio.2013.05.004 PMC3830630

[23] Grandin HM, Gemperli AC, Dard M (2012) Enamel matrix derivative: a review of cellular effects in vitro and a model of molecular arrangement and functioning. Tissue Eng Part B Rev 18: 181–202. 22070552 10.1089/ten.TEB.2011.0365

[24] Atsawasuwan P, Lu X, Ito Y, Zhang Y, Evans CA, et al (2013) Ameloblastin inhibits cranial suture closure by modulating MSX2 expression and proliferation. PLoS One 8: e52800. 23593111 10.1371/journal.pone.0052800 PMC3617155

[25] Hatakeyama J, Sreenath T, Hatakeyama Y, Thyagarajan T, Shum L, et al (2003) The receptor activator of nuclear factor-kappa B ligand-mediated osteoclastogenic pathway is elevated in amelogenin-null mice. J Biol Chem 278: 35743–35748. 12851394 10.1074/jbc.M306284200

[26] Gibson CW, Yuan ZA, Hall B, Longenecker G, Chen E, et al (2001) Amelogenin-deficient mice display an amelogenesis imperfecta phenotype. J Biol Chem 276: 31871–31875. 11406633 10.1074/jbc.M104624200

[27] Berdal A, Hotton D, Pike JW, Mathieu H, Dupret JM (1993) Cell- and stage-specific expression of vitamin D receptor and calbindin genes in rat incisor: regulation by 1,25-dihydroxyvitamin D3. Dev Biol 155: 172–179. 8380146 10.1006/dbio.1993.1016

[28] Nielsen Bs, Jorgensen S, Fog JU, Sokilde R, Christensen IJ, et al (2011) High levels of microRNA-21 in the stroma of colorectal cancers predict short disease-free survival in stage II colon cancer patients. Clin Exp Metastasis 28: 27–38. 21069438 10.1007/s10585-010-9355-7 PMC2998639

[29] Brookes SJ, Kirkham J, Shore RC, Wood SR, Slaby I, et al (2001) Amelin extracellular processing and aggregation during rat incisor amelogenesis. Arch Oral Biol 46: 201–208. 11165565 10.1016/s0003-9969(00)00121-7

[30] Uchida T, Murakami C, Dohi N, Wakida K, Satoda T, et al (1997) Synthesis, secretion, degradation, and fate of ameloblastin during the matrix formation stage of the rat incisor as shown by immunocytochemistry and immunochemistry using region-specific antibodies. J Histochem Cytochem 45: 1329–1340. 9313795 10.1177/002215549704501002

[31] Limeback H, Simic A (1989) Porcine high molecular weight enamel proteins are primarily stable amelogenin aggregates and serum albumin-derived proteins. In: ed. FR, editor. Tooth Enamel V. Yokohama, Japan: Florence Publishers. pp. 269–273.

[32] Jedeon K, De La Dure-Molla M, Brookes SJ, Loiodice S, Marciano C, et al (2013) Enamel defects reflect perinatal exposure to bisphenol A. Am J Pathol 183: 108–118. 23764278 10.1016/j.ajpath.2013.04.004 PMC3703547

[33] Aioub M, Lezot F, Molla M, Castaneda B, Robert B, et al (2007) Msx2 -/- transgenic mice develop compound amelogenesis imperfecta, dentinogenesis imperfecta and periodental osteopetrosis. Bone 41: 851–859. 17878071 10.1016/j.bone.2007.07.023

[34] Molla M, Descroix V, Aioub M, Simon S, Castaneda B, et al (2010) Enamel protein regulation and dental and periodontal physiopathology in MSX2 mutant mice. Am J Pathol 177: 2516–2526. 20934968 10.2353/ajpath.2010.091224 PMC2966808

[35] Haze A, Taylor AL, Blumenfeld A, Rosenfeld E, Leiser Y, et al (2007) Amelogenin expression in long bone and cartilage cells and in bone marrow progenitor cells. Anat Rec (Hoboken) 290: 455–460. 17393535 10.1002/ar.20520

[36] Spahr A, Lyngstadaas SP, Slaby I, Pezeshki G (2006) Ameloblastin expression during craniofacial bone formation in rats. Eur J Oral Sci 114: 504–511. 17184233 10.1111/j.1600-0722.2006.00403.x

[37] Athanassiou-Papaefthymiou M, Kim D, Harbron L, Papagerakis S, Schnell S, et al Molecular and circadian controls of ameloblasts. Eur J Oral Sci 119 Suppl 1: 35–40. 22243224 10.1111/j.1600-0722.2011.00918.x PMC3516856

[38] Zheng L, Seon YJ, Mourao MA, Schnell S, Kim D, et al Circadian rhythms regulate amelogenesis. Bone 55: 158–165. 23486183 10.1016/j.bone.2013.02.011 PMC3650122

[39] Sodek J, McKee MD (2000) Molecular and cellular biology of alveolar bone. Periodontol 2000 24: 99–126. 11276877 10.1034/j.1600-0757.2000.2240106.x

[40] Atsawasuwan P, Lu X, Ito Y, Chen Y, Gopinathan G, et al (2013) Expression and function of enamel-related gene products in calvarial development. J Dent Res 92: 622–628. 23625374 10.1177/0022034513487906 PMC3684230

[41] Alikhani M, Khoo E, Alyami B, Raptis M, Salgueiro JM, et al (2012) Osteogenic effect of high-frequency acceleration on alveolar bone. J Dent Res 91: 413–419. 22337699 10.1177/0022034512438590 PMC3310758

[42] Tamburstuen MV, Reppe S, Spahr A, Sabetrasekh R, Kvalheim G, et al (2010) Ameloblastin promotes bone growth by enhancing proliferation of progenitor cells and by stimulating immunoregulators. Eur J Oral Sci 118: 451–459. 20831578 10.1111/j.1600-0722.2010.00760.x

[43] Zhou YL, Snead ML (2000) Identification of CCAAT/enhancer-binding protein alpha as a transactivator of the mouse amelogenin gene. J Biol Chem 275: 12273–12280. 10766866 10.1074/jbc.275.16.12273

[44] Fukumoto S, Kiba T, Hall B, Iehara N, Nakamura T, et al (2004) Ameloblastin is a cell adhesion molecule required for maintaining the differentiation state of ameloblasts. J Cell Biol 167: 973–983. 15583034 10.1083/jcb.200409077 PMC2172447

[45] Sonoda A, Iwamoto T, Nakamura T, Fukumoto E, Yoshizaki K, et al (2009) Critical role of heparin binding domains of ameloblastin for dental epithelium cell adhesion and ameloblastoma proliferation. J Biol Chem 284: 27176–27184. 19648121 10.1074/jbc.M109.033464 PMC2785645

[46] Rached MT, Kode A, Silva BC, Jung DY, Gray S, et al (2010) FoxO1 expression in osteoblasts regulates glucose homeostasis through regulation of osteocalcin in mice. J Clin Invest 120: 357–368. 20038793 10.1172/JCI39901 PMC2798687

[47] Beyeler M, Schild C, Lutz R, Chiquet M, Trueb B (2010) Identification of a fibronectin interaction site in the extracellular matrix protein ameloblastin. Exp Cell Res 316: 1202–1212. 20043904 10.1016/j.yexcr.2009.12.019

[48] Wang H, Tannukit S, Zhu D, Snead ML, Paine ML (2005) Enamel matrix protein interactions. J Bone Miner Res 20: 1032–1040. 15883644 10.1359/JBMR.050111

[49] Coxon TL, Brook AH, Barron MJ, Smith RN (2012) Phenotype-genotype correlations in mouse models of amelogenesis imperfecta caused by Amelx and Enam mutations. Cells Tissues Organs 196: 420–430. 22759786 10.1159/000336440 PMC3718574

[50] Wazen RM, Moffatt P, Zalzal SF, Yamada Y, Nanci A (2009) A mouse model expressing a truncated form of ameloblastin exhibits dental and junctional epithelium defects. Matrix Biol 28: 292–303. 19375505 10.1016/j.matbio.2009.04.004 PMC2727877

[51] Kuroda S, Wazen R, Sellin K, Tanaka E, Moffatt P, et al (2011) Ameloblastin is not implicated in bone remodelling and repair. Eur Cell Mater 22: 56–66 discussion 66–57. 21761392 10.22203/ecm.v022a05

[52] Smith CE, Wazen R, Hu Y, Zalzal SF, Nanci A, et al (2009) Consequences for enamel development and mineralization resulting from loss of function of ameloblastin or enamelin. Eur J Oral Sci 117: 485–497. 19758243 10.1111/j.1600-0722.2009.00666.x PMC2778578

[53] Kitagawa M, Kitagawa S, Nagasaki A, Miyauchi M, Uchida T, et al (2011) Synthetic ameloblastin peptide stimulates differentiation of human periodontal ligament cells. Arch Oral Biol 56: 374–379. 21074142 10.1016/j.archoralbio.2010.10.012

[54] Zhang Y, Zhang X, Lu X, Atsawasuwan P, Luan X (2011) Ameloblastin regulates cell attachment and proliferation through RhoA and p27. Eur J Oral Sci 119 Suppl 1: 280–285. 22243257 10.1111/j.1600-0722.2011.00887.x PMC3402544

[55] Huang YC, Tanimoto K, Tanne Y, Kamiya T, Kunimatsu R, et al (2010) Effects of human full-length amelogenin on the proliferation of human mesenchymal stem cells derived from bone marrow. Cell Tissue Res 342: 205–212. 20967466 10.1007/s00441-010-1064-7

[56] Wen X, Cawthorn WP, MacDougald OA, Stupp SI, Snead ML, et al (2011) The influence of Leucine-rich amelogenin peptide on MSC fate by inducing Wnt10b expression. Biomaterials 32: 6478–6486. 21663957 10.1016/j.biomaterials.2011.05.045 PMC3134126

[57] Zeichner-David M, Chen LS, Hsu Z, Reyna J, Caton J, et al (2006) Amelogenin and ameloblastin show growth-factor like activity in periodontal ligament cells. Eur J Oral Sci 114 Suppl 1: 244–253; discussion 254–246, 381–242 16674693 10.1111/j.1600-0722.2006.00322.x

[58] Fukae M, Kanazashi M, Nagano T, Tanabe T, Oida S, et al (2006) Porcine sheath proteins show periodontal ligament regeneration activity. Eur J Oral Sci 114 Suppl 1: 212–218; discussion 254–216, 381–212 16674688 10.1111/j.1600-0722.2006.00309.x

[59] Matsuzawa M, Sheu TJ, Lee YJ, Chen M, Li TF, et al (2009) Putative signaling action of amelogenin utilizes the Wnt/beta-catenin pathway. J Periodontal Res 44: 289–296. 19462488 10.1111/j.1600-0765.2008.01091.x

[60] Kakegawa A, Oida S, Gomi K, Nagano T, Yamakoshi Y, et al (2010) Cytodifferentiation activity of synthetic human enamel sheath protein peptides. J Periodontal Res 45: 643–649. 20572923 10.1111/j.1600-0765.2010.01279.x

[61] Kunimatsu R, Tanimoto K, Tanne Y, Kamiya T, Ohkuma S, et al (2011) Amelogenin enhances the proliferation of cementoblast lineage cells. J Periodontol 82: 1632–1638. 21486179 10.1902/jop.2011.110031

[62] Izumikawa M, Hayashi K, Polan MA, Tang J, Saito T (2012) Effects of amelogenin on proliferation, differentiation, and mineralization of rat bone marrow mesenchymal stem cells in vitro. ScientificWorldJournal 2012: 879731. 22547998 10.1100/2012/879731 PMC3322511

[63] Amin HD, Olsen I, Knowles JC, Dard M, Donos N (2013) Effects of enamel matrix proteins on multi-lineage differentiation of periodontal ligament cells in vitro. Acta Biomater 9: 4796–4805. 22985741 10.1016/j.actbio.2012.09.008

[64] Amin HD, Olsen I, Knowles JC, Donos N (2012) Differential effect of amelogenin peptides on osteogenic differentiation in vitro: identification of possible new drugs for bone repair and regeneration. Tissue Eng Part A 18: 1193–1202. 22320389 10.1089/ten.TEA.2011.0375

[65] Fincham AG, Moradian-Oldak J, Simmer JP, Sarte P, Lau EC, et al (1994) Self-assembly of a recombinant amelogenin protein generates supramolecular structures. J Struct Biol 112: 103–109. 8060728 10.1006/jsbi.1994.1011

[66] Wald T, Osickova A, Sulc M, Benada O, Semeradtova A, et al (2013) Intrinsically disordered enamel matrix protein ameloblastin forms ribbon-like supramolecular structures via an N-terminal segment encoded by exon 5. J Biol Chem 288: 22333–22345. 23782691 10.1074/jbc.M113.456012 PMC3829324

[67] Bartlett Jd, Skobe Z, Lee Dh, Wright Jt, Li Y, et al (2006) A developmental comparison of matrix metalloproteinase-20 and amelogenin null mouse enamel. Eur J Oral Sci 114 Suppl 1: 18–23; discussion 39–41, 379 16674657 10.1111/j.1600-0722.2006.00292.x

[68] Simmer JP, Hu CC, Lau EC, Sarte P, Slavkin HC, et al (1994) Alternative splicing of the mouse amelogenin primary RNA transcript. Calcif Tissue Int 55: 302–310. 7820782 10.1007/BF00310410

[69] Li W, Mathews C, Gao C, DenBesten PK (1998) Identification of two additional exons at the 3′ end of the amelogenin gene. Arch Oral Biol 43: 497–504. 9717587 10.1016/s0003-9969(98)00013-2

[70] Lau EC, Simmer JP, Bringas P Jr, Hsu DD, Hu CC, et al (1992) Alternative splicing of the mouse amelogenin primary RNA transcript contributes to amelogenin heterogeneity. Biochem Biophys Res Commun 188: 1253–1260. 1445358 10.1016/0006-291x(92)91366-x

