# Supplementary figures and images for: Tracking Endogenous Amelogenin and Ameloblastin In Vivo
Source: PLoS One. 2014 Jun 16;9(6):e99626. doi: 10.1371/journal.pone.0099626 (PMC4059656; doi:10.1371/journal.pone.0099626)

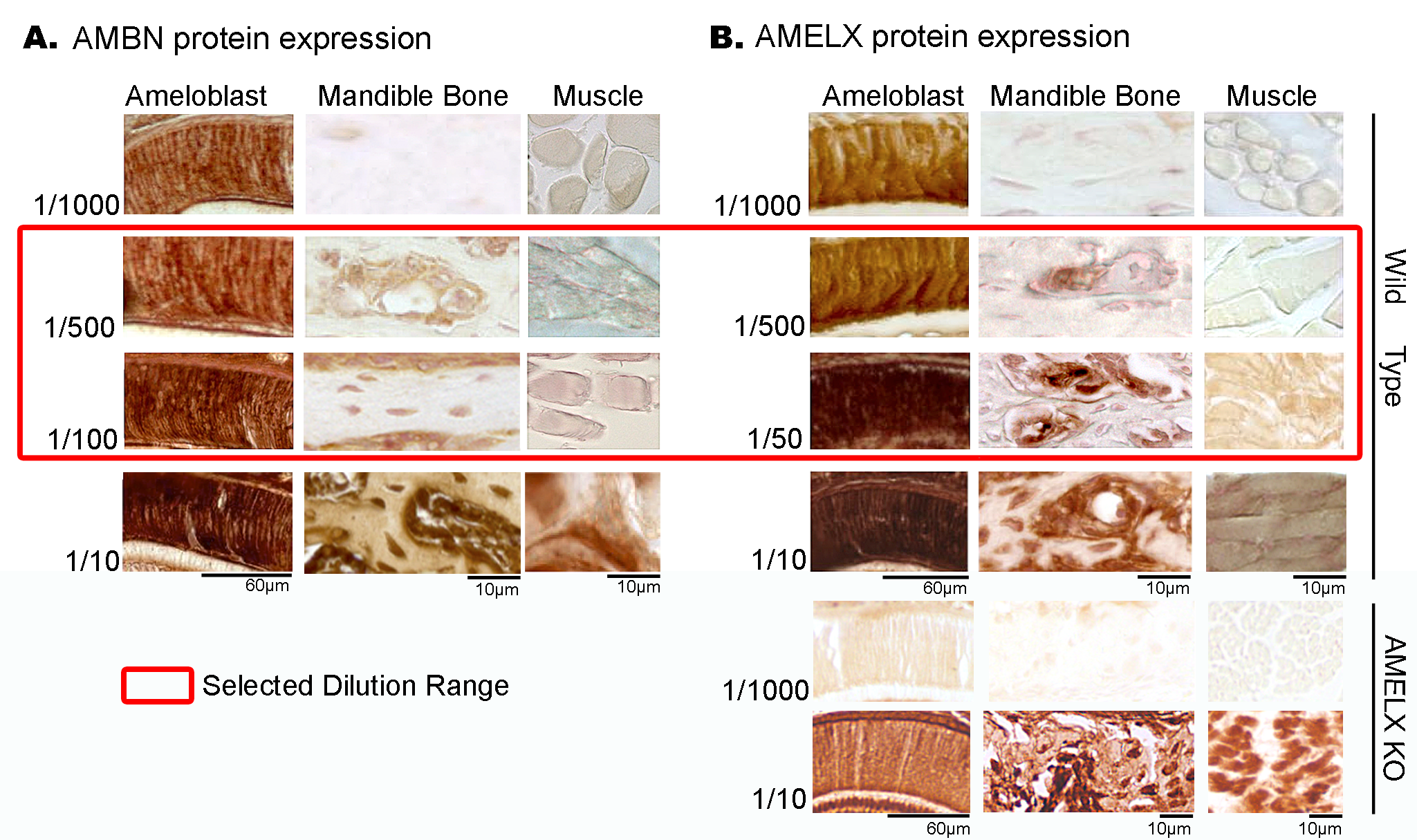

Supplement: Figure S1 — Optimization of AMBN and AMELX immunodetection in 1 week old WT and AMELX KO mice. A-B. When using a classic dilution of antibody (1/1,000), cross reactivity to AMELX and AMBN proteins is restricted to the dental epithelial cells (EP) (positive control tissue) in WT mouse, a pattern well documented in the literature. Dilutions of 1/500 show the presence of AMBN and AMELX protein expression in EP, but also in some bone stromal cells. Increased concentration of antibody (1/100 for AMBN and 1/50 for AMELX) results in stronger staining in osteoblasts, in osteocytes and in mandibular bone matrix. At dilution 1/100 (AMBN) or 1/50 (AMELX), striated muscle (negative control tissue) shows diffuse staining indicating the limit of specificity for these antibodies. At dilution 1/10, the striated muscle was clearly stained for both AMBN and AMELX, determining the non-specificity threshold. This threshold was confirmed using AMELX KO mouse as a bona fide control, non-specific cross reactivity signal being observed when using 1/10 antibody dilution. For detailed immunohistoperoxidase methods see File S1. (TIF) [file pone.0099626.s001.tif]

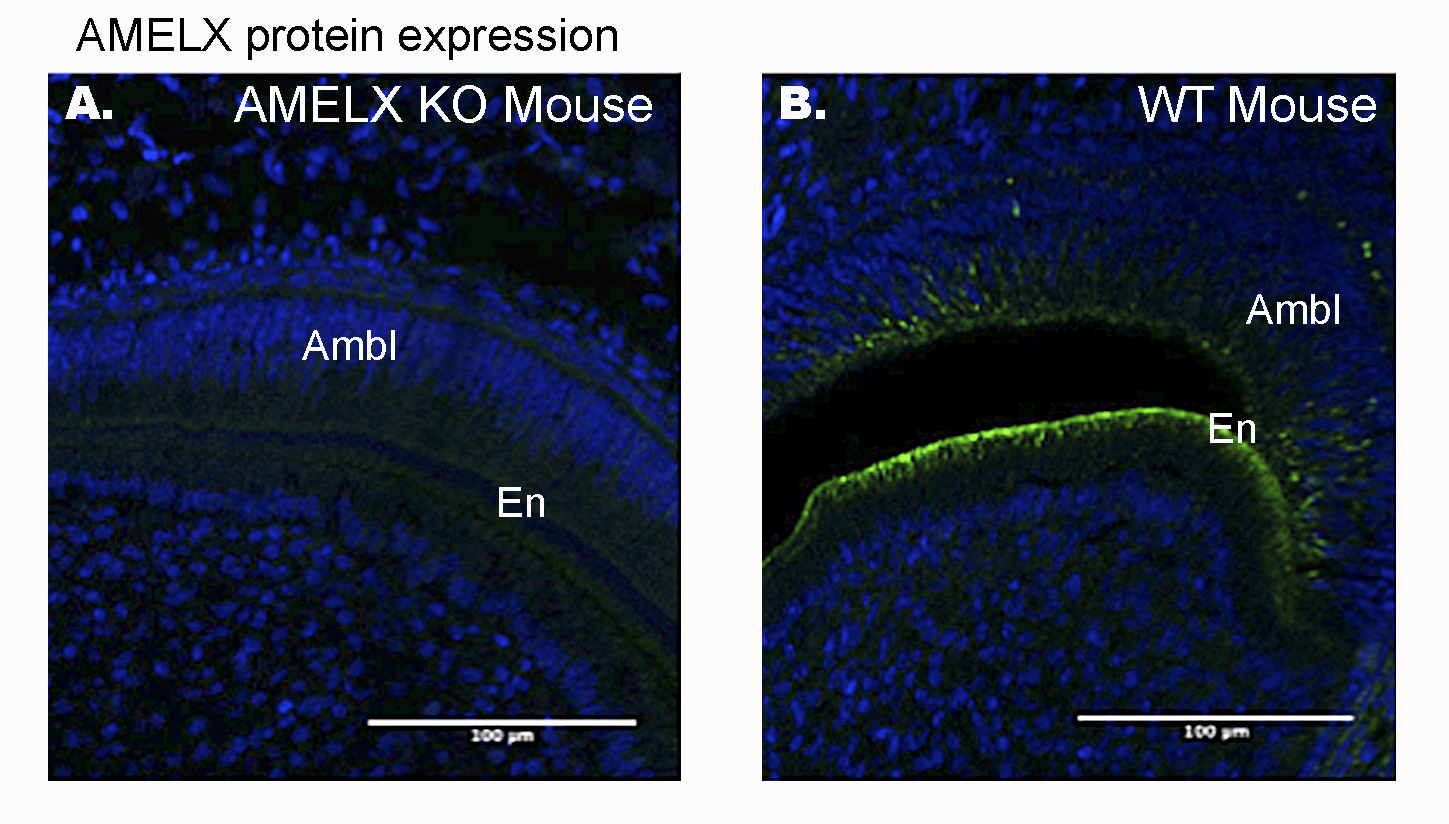

Supplement: Figure S2 — AMELX protein expression in 1 week old WT and AMELX KO mice. A. When using 1/500 antibody dilution, AMELX (green staining) is not detected in AMELX KO mice. B. Using the same antibody dilution, WT section show anti-AMELX cross reactivity in enamel (En) and ameloblasts (Ambl). (TIF) [file pone.0099626.s002.tif]

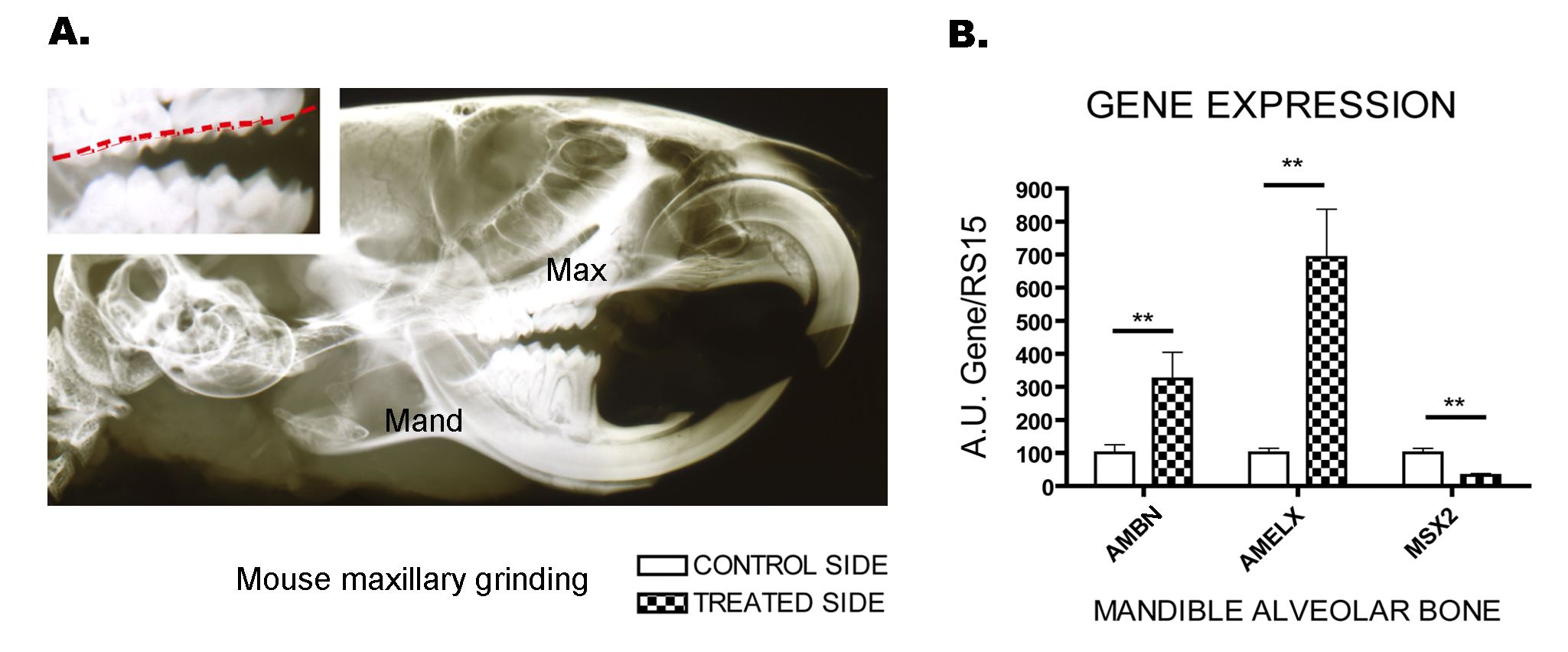

Supplement: Figure S3 — Impact of mechanical stimuli on AMBN and AMELX mRNA expression in jaw. A. Right maxillary molar cusps in 15 week old WT mice were ground flat to the level indicated by the red dotted line. B. 72 h after grinding, significant increase in mRNA levels of AMELX (x7) and AMBN (x3) is observed in treated mandible alveolar bone. Concurrently, a significant reduction in MSX2 mRNA level (x3) is observed; MSX2 being a transcriptional repressor of AMELX gene. mRNA expression of those genes are unaffected in basal bone (data not shown). mRNA levels of genes of interest are normalized against mRNA expression of the housekeeping gene RS15 (F5′-ggcttgtaggtgatggagaa-3′/R5′-cttccgcaagttcacctacc-3′). Overall probability was determined using KW test (**p<0.05). For detailed molar grinding and flattening procedures see File S1. (TIF) [file pone.0099626.s003.tif]
