# Supplementary material for: Tracking Endogenous Amelogenin and Ameloblastin In Vivo
Source: PLoS One. 2014 Jun 16;9(6):e99626. doi: 10.1371/journal.pone.0099626 (PMC4059656; doi:10.1371/journal.pone.0099626)
Supplement: File S1 — Detailed procedures. (DOCX) [file pone.0099626.s004.docx]

# Supporting information

**Immunohistoperoxidase.** Tissues were collected from 1 week old WT and AMELX KO mice and prepared as described in the main manuscript. Primary anti-AMBN antibody (sc 50534 (M-300), Santa Cruz Biotechnology) and anti-AMELX antibody (ab 59705, Abcam) diluted from 1:10 to 1:1,000 were used.

**Molar grinding and flattening**

15 week old WT mice were anesthetized with intraperitoneal injection of Rompun (Xylasine 15 mg/kg) and Imalgene (Ketamine 100 mg/kg). Cusps of the maxillary right molars were ground down and carefully flattened with a diamond burr mounted on a handpiece to avoid exposing the pulp chamber. Alveolar bone and basal bone from treated (right hemi mandible) and control (left hemi mandible) sides were microdissected 72 h after treatment. RNA was extracted and qRT-PCR performed as described in the manuscript.
